# Poor medical student welfare—a growing threat to healthcare

**DOI:** 10.1186/s43045-021-00135-3

**Published:** 2021-08-20

**Authors:** Andrew Molodynski, Sarah Marie Farrell, Dinesh Bhugra, Tarek Okasha

**Affiliations:** 1 Oxford Health NHS Foundation Trust, Oxford, UK; 2 Oxford University, Oxford, UK; 3 University College Hospital, London, UK; 4 King’s College, London, UK; 5 Okasha Institute of Psychiatry, Cairo, Egypt

The events of the last year have highlighted the importance of healthcare systems and of those who work in them to the wellbeing and functioning of society. As SARS-CoV2 has swept around the world, touching every family in every country and causing millions of deaths directly, scenes of exhausted health workers in overwhelmed hospitals have been seen in many countries. High-income countries have for once been severely affected, and social and economic inequalities in those societies have been brought into sharp relief by significantly higher death rates amongst the poor, the mentally ill, and those from ethnic minority groups. As we enter the longer-term phase of ‘COVID management’, issues are arising regarding the fair distribution of vaccines (so-called ‘vaccine nationalism’) and the need for ongoing supplies of protective and medical equipment.

In amongst all of this, there has been universal recognition and applause (often literally) for health workers, including doctors. COVID has reminded us, if indeed a reminder was needed, that however well or poorly resourced a health care system is, it is the people who work in it who are the most precious resource. This resource needs looking after. Wellbeing support for healthcare staff remains poor in general and minimal or absent in many countries. This sometimes relates to lack of finance, but also is a consequence of a lack of awareness and/or lack of prioritisation. Things are now changing significantly. Just as COVID has accelerated the role of digital medicine by a number of years, it has shone a light on poor working conditions and low levels of welfare amongst health staff. Interventions to improve welfare will need to be tailored to the different needs of different disciplines, with fair wages and adequate protective equipment being important for lower-paid staff especially. For doctors, there is evidence of an increasing need for supportive work environments, peer support, access to help when needed, and adequate facilities to provide good quality health care.

The pandemic has heightened the role of medical students in health care provision, with many drafted in and given early registration to support front-line services internationally. This has highlighted the stress they face and their need for adequate support that protects their welfare and psychological wellbeing as well as equipping them to be effective and compassionate doctors over long careers. This is important for the students themselves but also for the welfare of future generations of patients.

The pandemic has helped to usher in a new age of digital and remote medicine, much of which is welcome. However, there must be continuing efforts to refine technology to improve the accessibility and quality of care. It also has underlined that despite this we will always need human beings to deliver care, whatever the format. The fundamental importance of interpersonal relationships in healthcare has been highlighted by the tragic situations where patients have died without being able to be held by or even see the faces of relatives and staff. Any illusions that politicians may have had, and they were illusions, that technology would radically reduce the need for doctors, have been removed. In fact, we know that we will need ever-increasing numbers of doctors to continue to deliver care to a global population of 7.64 billion people (and growing fast), whilst leading on the development of healthcare provision and the response to crises (https://data.worldbank.org/indicator/SP.POP.TOTL).

Despite an increasing focus on the wellbeing of the medical workforce globally, the picture remains grim. A large-scale study of 3766 medical students in 12 countries across five continents last year reported extremely high rates of burnout, mental ill health, and substance misuse amongst medical students [2]. Three quarters of medical students screened as having a diagnosable mental health problem using the General Health Questionnaire-12 ( [1]) and 78% were disengaged and 87% exhausted using the Oldenburg Burnout Inventory [3] respectively. Almost one in eight students screened positive for substance misuse using validated tools. Numerous stressors were identified in various key aspects of life such as housing, finances, relationships, and academic performance.

The accumulating evidence suggests that these high rates of burnout and mental health problems will continue as students become doctors in training and then become senior doctors. They may well worsen, and research clearly shows high rates of difficulties in those groups [4]. If we can act early by supporting these doctors of the future from the moment they enter training, then we may be able to reduce the burden of distress and learn how and when to intervene when it occurs.

Over the next few editions of Middle East Current Psychiatry, reports from a number of countries around the world will be presented that further highlight ‘where we are now’ in regard to student welfare at this pivotal time in modern medical history. This knowledge needs to form the basis for urgent action to improve student welfare- for the students, themselves, for society, and for those who will need them for generations to come.
